# Urine Matrix Metalloproteinase-7 (MMP-7) Versus Urine Albumin-to-Creatinine Ratio (ACR) as Predictors of Renal Dysfunction: A Decision Curve Analysis

**DOI:** 10.7759/cureus.78275

**Published:** 2025-01-31

**Authors:** Rajlaxmi Sarangi, Debadyuti Sahu, Nikunj Kishore Rout, Krishna Padarabinda Tripathy, Saurav Patra, Jyotirmayee Bahinipati, Jyoti Prakash Sahoo

**Affiliations:** 1 Biochemistry, Kalinga Institute of Medical Sciences, Bhubaneswar, IND; 2 Nephrology, Kalinga Institute of Medical Sciences, Bhubaneswar, IND; 3 General Medicine, Kalinga Institute of Medical Sciences, Bhubaneswar, IND; 4 Pharmacology, Kalinga Institute of Medical Sciences, Bhubaneswar, IND

**Keywords:** albumin-to-creatinine ratio, blood creatinine level, chronic renal failure, decision curve analysis, diabetic kidney injury, matrix metalloproteinase, net benefit, predictive modeling, receiver operating characteristic curve (roc), threshold probability

## Abstract

Background and objectives: The level of matrix metalloproteinase-7 (MMP-7) in diabetic urine samples escalates owing to reduced renal function. Renal biopsy is rarely recommended due to its invasive nature. Nowadays, urine albumin-to-creatinine ratio (ACR) is widely used to assess renal impairment. We mapped this study to compare urine MMP-7 and urine ACR as indicators of renal impairment.

Methods: This cross-sectional study was conducted at Kalinga Institute of Medical Sciences (KIMS), Bhubaneswar, India, from February 2020 to January 2023. Adult patients with either type 2 diabetes mellitus (T2DM), kidney disease, or hypertension were scrutinized. Their serum creatinine, urine albumin, urine creatinine, urine ACR, and urine MMP-7 levels were evaluated. We correlated serum creatinine values with urine ACR and urine MMP-7 levels. For predictive modeling, we developed two models: ACR_model and MMP7_model. The ACR_model and MMP7_model weighed renal activity by analyzing participants with urine ACR > 30 mg/g and normalized urine MMP-7 > 10 µg/L, respectively. Each model's predictive accuracy was computed using the area under the receiver-operating characteristic (ROC) curve (AUC). For predictive modeling, we deployed the bootstrap method and decision curve analysis. We used R software (version 4.4.2) for data analysis.

Results: A total of 287 (87.5%) of the 328 patients we scrutinized were deemed eligible for the study. We found statistically significant correlation coefficients of serum creatinine with urine MMP-7 than with urine ACR. It suggested a stronger association of serum creatinine with urine MMP-7. The study revealed that urine ACR had lower values of the following parameters than urine MMP-7: sensitivity (81.3% versus 86.7%), specificity (64.4% versus 68.2%), and diagnostic accuracy (78.2% versus 86.6%). The decision curve analysis unveiled that urine MMP-7 demonstrated higher net benefits juxtaposed with urine ACR, regardless of the threshold probability.

Conclusion: This study analyzed the role of urine ACR and urine MMP-7 as biomarkers of renal failure. We discovered stronger correlations between serum creatinine and urine MMP-7 contrasted with urine ACR. Urine MMP-7 offered better specificity, sensitivity, and diagnostic accuracy than urine ACR. The decision curve analysis also revealed that urine MMP-7 outperformed urine ACR in forecasting renal impairment.

## Introduction

Matrix metalloproteinase (MMP) degrades the extracellular matrix (ECM). The most important version, matrix metalloproteinase-7 (MMP-7), relies on calcium and zinc for its activity [1]. Nephropathy is a frequent microvascular consequence in diabetic people [2,3]. Diabetic kidney disease (DKD) commences with glomerular basement membrane (GBM) thickening prompted by aberrant ECM deposits [4-6]. DKD causes dysregulation of the gremlin-1 (BMP) pathway and the renin-angiotensin-aldosterone system (RAAS). DKD patients had higher urinary excretions of angiotensinogen, gremlin-1, and MMP-7 due to abnormalities in these pathways [4,7].

Advanced glycation end products (AGEs) expedite DKD progression [8]. Recent studies indicate a link between increased levels of urine MMP-7 in DKD and AGEs [9-11]. Because MMP-7 frequently transpires in the renal tubular epithelium, DKD patients experience altered renal MMP-7 expression levels [10,12]. MMP-7 urine excretion appears to be a better indicator than serum MMP-7 in patients with kidney damage, despite their diabetic record [12]. As an illustration, urine MMP-7 testing yields a clearer picture of kidney health than serum MMP-7. However, the urine MMP-7 level must be balanced with the urine creatinine level [12,13].

Recently, a few papers revealed some cases of DKD with no evidence of microalbuminuria and a lower estimated glomerular filtration rate (eGFR) [14-16]. Renal biopsy is a highly precise diagnostic method for distinguishing DKD from other kidney diseases. Nonetheless, it is only seldom suggested, pertaining to its invasive nature [17]. Another method for gauging renal function is to calculate the urine albumin-to-creatinine ratio (ACR). Higher urine ACR values indicate significant renal impairment [18,19]. Our earlier research paper compared several glycemic and renal parameters in patients with or without type 2 diabetes mellitus (T2DM) and renal dysfunction [20]. This article investigated the relationship between serum creatinine, urine MMP-7, and urine ACR in study participants. We also employed the receiver operating characteristic (ROC) curve and decision curve analysis to use urine MMP-7 and ACR values to predict renal impairment.

## Materials and methods

The cross-sectional study spanned from February 2020 to January 2023. Before undertaking our investigation, we received ethical approval (KIIT/KIMS/IEC/211/2020) from the Institutional Ethics Committee of Kalinga Institute of Medical Sciences (KIMS), Bhubaneswar, India. Before beginning the enrollment process, each participant gave written informed consent.

Study criteria

We included adult patients diagnosed with T2DM, renal illness, or hypertension. Kidney transplant patients, those on hemodialysis, those receiving treatment for diabetic retinopathy, and those with autoimmune or infectious diseases affecting the kidneys, malignancies, rheumatoid arthritis, and thromboembolic events within the previous six months were excluded from the study. We also excluded nursing mothers and pregnant women.

Study design and objectives

The subjects in this cross-sectional study were grouped based on their blood sugar level and renal function. We skipped over hypertension in the group selection procedure. Group A included people with both T2DM and renal impairment. Group B comprised diabetic subjects without kidney disease. Group C encompassed individuals without T2DM and renal disease. Group D patients exhibited kidney illness but did not have T2DM.

In the current research, we probed the correlation of urine MMP-7 and urine ACR with serum creatinine of the study participants. We employed receiver operating characteristic (ROC) curves to compare the sensitivity and specificity of urine MMP-7 to that of urine ACR in assessing renal function. Through the decision curve analysis with urine MMP-7 and urine ACR models, we did the predictive modeling of renal deterioration.

Study procedure

From each participant, 3 mL of blood and 5 mL of first-morning midstream urine samples were taken. For fasting blood sugar (FBS) measurement, 1 mL of the 3 mL blood sample was stored in a fluorine vial. An additional 1 mL of blood was stored in an ethylene-diamine tetraacetic acid (EDTA) vial to measure glycated hemoglobin (HbA_1C_). The remaining 1 mL sample was preserved in a red-top vacutainer for serum separation. Centrifugation was done for 10 minutes at 3000 RPM following clot retraction. The quantitative serum creatinine and urea estimations were carried out using DxC 700 AU Beckman Coulter autoanalyzer (Brea, CA).

Of the 5 mL urine sample, 1 mL was tested to quantify urine albumin and creatinine levels. The microalbumin and creatinine were estimated in the DxC 700 AU Beckman Coulter autoanalyzer^®^. The urine ACR was expressed as mg of albumin in urine per gram of creatinine excreted. The remaining 4 mL of urine was centrifuged at 2000-3000 RPM for 20 minutes. For MMP-7 estimation, 100 μL of supernatant was stored at -80°C using an enzyme-linked immunosorbent assay (ELISA). Urine MMP-7 levels were quantified weekly using a RayBio^®^ Human MMP-7 ELISA kit (catalog number: ELH-MMP7) (Norcross, GA). The normalized urine MMP-7 levels were calculated by dividing urine MMP-7 by urine creatinine. We deployed the modification of diet in renal disease (MDRD) formula to classify eGFR [21]. Leveraging the MDRD formula (eGFR = 175 × SCr-1.154 × age-0.203 × 0.742 (if female) × 1.21 (if Black)), the kidney disease staging was performed as follows: stage I: eGFR of ≥90 mL/minute/1.73 m²; stage II: eGFR of 60-89 mL/minute/1.73 m²; stage III: eGFR of 30-59 mL/minute/1.73 m²; stage IV: eGFR of 15-29 mL/minute/1.73 m²; and stage V: eGFR of <15 mL/minute/1.73 m². We considered stages IV and V as kidney disease in this study.

We used urine MMP-7 and ACR levels to estimate our research participants' renal function. Two models were developed for predictive modeling: ACR_model and MMP7_model. The ACR_model and MMP7_model predicted renal function by evaluating individuals with urine ACR > 30 mg/g and normalized urine MMP-7 > 10 µg/L, respectively. Each model's predictive accuracy was computed using the area under the ROC curve (AUC). The bootstrap approach was used to ascertain the predictive modeling.

Sample size calculation

For calculating the sample size, we adopted mean proportions of 0.6 and 0.4 for renal disease among diabetic and nondiabetic persons, respectively. A sample of 258 subjects was required, considering beta and two-sided alpha errors of 0.10 and 0.05, respectively. We obtained a sample size of 277 after deploying the Fleiss correction for continuity. The final study, however, encompassed 287 subjects (169 with and 118 without T2DM).

Statistical analysis

We verified the normality of the data distribution through the Shapiro-Wilk test and spotted it as non-parametric. Qualitative data were summarized using frequency and percentage. Quantitative data were represented using median and interquartile range (IQR). We analyzed qualitative and quantitative data using Pearson's chi-square and Kruskal-Wallis tests, respectively. We utilized pROC [22] and rmda [23] packages in R (version 4.4.2) [24] for data analysis. Statistical significance was defined as p-values < 0.05.

## Results

The cross-sectional study was conducted from February 2020 to January 2023. For this study, we scrutinized 328 patients. Thirty-four of them declined to participate, and seven were not adults. Two hundred eighty-seven people were segregated into four groups per their glycemic and renal statuses. Their demographic and clinical traits are presented in Table 1. Group A consisted of 94 patients with T2DM and renal disease. Group B consisted of 75 diabetics without kidney disease. Group C entailed 65 people without T2DM and renal disease. Group D contained 53 nondiabetic persons with renal dysfunction. The participants had a median age of 52.0 (44.0-61.1) years. The median value for serum creatinine was 1.97 (0.81-2.48) mg/dL. The median urine ACR of the study population was 151.2 (10.7-199.1) mg/g. Normalized urine MMP-7 values ranged from 1.1 to 50.5 µg/L, with 19.9 µg/L as median. Most of the demographic attributes and clinical parameters varied significantly across groups. The presence of T2DM and kidney impairment could explain these disparities.

**Table 1:** Demographic and clinical traits of the study population Median with IQR was chosen to depict continuous variables. Frequency and percentage were the measures used to display categorical variables. The continuous and categorical variables were gauged with the Kruskal-Wallis and chi-square (ꭓ2) tests. Groups A, B, C, and D entailed individuals with diabetic nephropathy, diabetes only, without diabetes and nephropathy, and nephropathy only, respectively. BMI: body mass index, eGFR: estimated glomerular filtration rate, urine ACR: urine albumin-to-urine creatinine ratio, MMP-7: matrix metalloproteinase-7, normalized urine MMP-7: urine MMP-7-to-urine creatinine ratio, IQR: interquartile range

Parameters	Total (N = 287)	Group A (n = 94)	Group B (n = 75)	Group C (n = 65)	Group D (n = 53)	Test statistics	p-value
Age (years)	52.0 (44.0-61.0)	57.5 (49.0-63.0)	47.0 (40.5-55.0)	44.0 (30.0-52.0)	56.0 (51.0-63.0)	829.8	<0.001
Age group							
<40 years	44 (15.3%)	3 (3.2%)	12 (16.0%)	29 (44.6%)	0	154.3	0.808
40-60 years	170 (59.2%)	55 (58.5%)	53 (70.7%)	28 (43.1%)	34 (64.2%)		
>60 years	73 (25.5%)	36 (38.3%)	10 (13.3%)	8 (12.3%)	19 (35.8%)		
Males (%)	173 (60.3%)	68 (72.3%)	38 (50.7%)	35 (53.8%)	32 (60.4%)	149.8	0.812
BMI (kg/m^2^)	24.2 (22.3-25.2)	22.1 (20.9-24.2)	24.4 (23.2-25.1)	24.5 (23.7-25.9)	24.4 (23.8-25.4)	52.9	0.931
Urea (mg/dL)	41.0 (10.0-56.0)	58.0 (48.0-62.0)	11.0 (10.5-12.0)	9.0 (8.0-10.0)	55.0 (51.0-62.0)	4744.4	<0.001
Serum creatinine (mg/dL)	1.97 (0.81-2.48)	2.65 (2.18-2.86)	0.81 (0.73-0.87)	0.81 (0.72-0.84)	2.34 (2.17-2.57)	639.7	<0.001
eGFR (mL/minute/m^2^)	35.7 (24.2-92.3)	23.7 (21.5-26.1)	90.6 (84.1-96.0)	95.7 (87.8-105.9)	24.5 (23.2-29.9)	6014.0	<0.001
Urine albumin (µg/dL)	3.38 (1.11-4.23)	4.83 (4.14-5.20)	1.22 (1.11-1.34)	1.04 (0.97-1.09)	3.77 (3.53-4.12)	603.8	0.024
Urine creatinine (mg/dL)	35.8 (20.5-111.9)	24.5 (18.8-30.4)	88.9 (67.9-101.5)	173.6 (154.2-198.6)	16.9 (14.7-20.1)	16811.0	<0.001
Urine ACR (mg/g)	151.2 (10.7-199.1)	163.7 (156.9-277.2)	13.7 (11.9-17.4)	6.1 (5.3-6.7)	243.4 (168.8-255.9)	29994.0	<0.001
Serum Na^+^ (mg/dL)	140.0 (137.8-142.9)	140.1 (137.5-143.9)	140.2 (138.0-142.5)	140.0 (137.9-142.8)	139.8 (137.3-142.9)	54.7	0.914
Serum K^+^ (mg/dL)	4.57 (4.07-4.79)	4.62 (4.16-4.94)	4.44 (3.95-4.65)	4.48 (4.25-4.66)	4.91 (3.77-5.10)	20.7	0.967
Normalized urine MMP-7 levels (µg/L)	19.9 (1.1-50.5)	64.4 (51.8-89.6)	1.4 (1.1-1.8)	0.9 (0.7-1.1)	34.5 (23.5-40.4)	10745.0	<0.001

**Figure 1:**
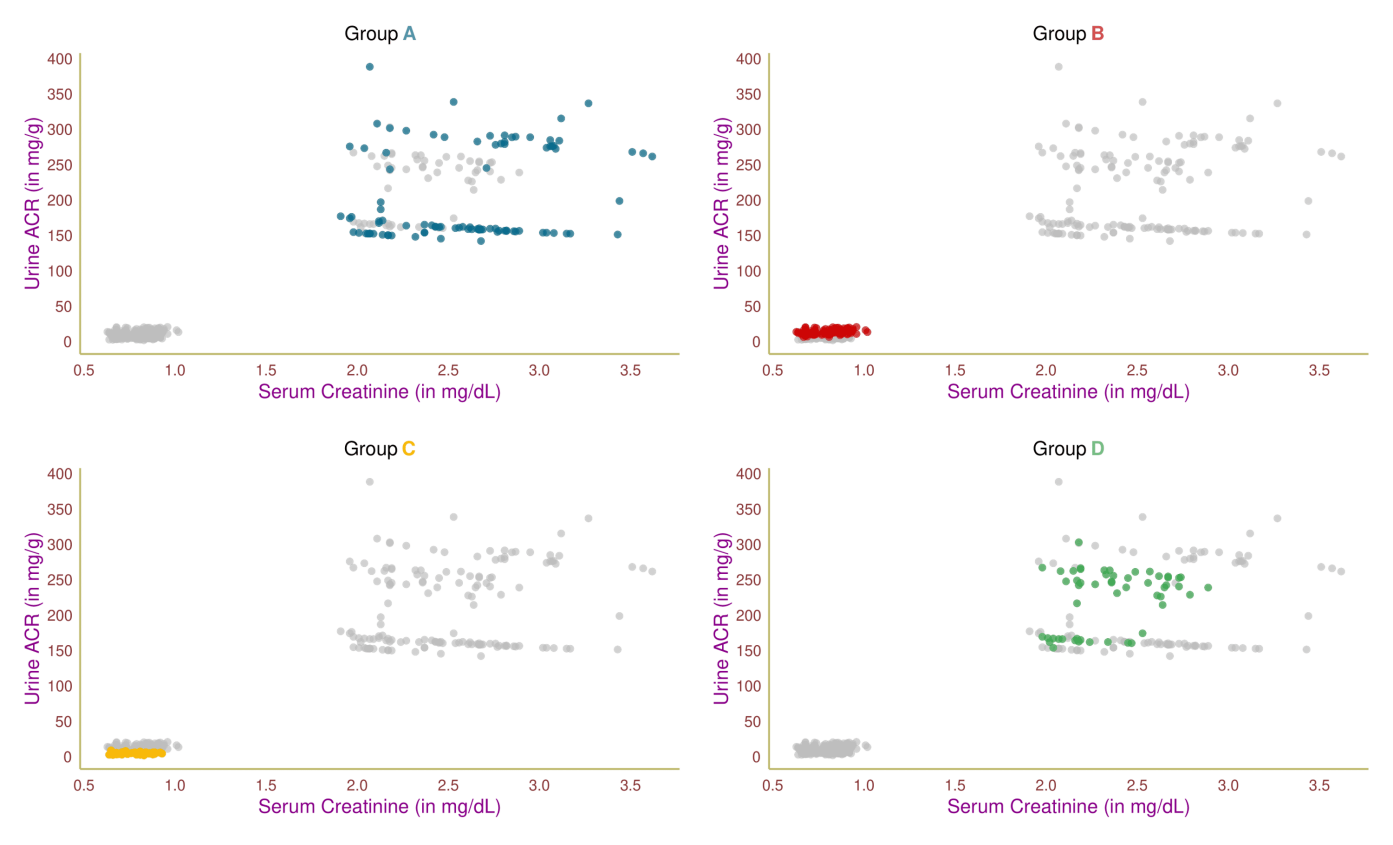
Association between serum creatinine and urine ACR The scatterplots showcase the association between serum creatinine and urine ACR values of the study participants in different colors. Groups A, B, C, and D encompassed individuals with diabetic nephropathy, diabetes only, without diabetes and nephropathy, and nephropathy only, respectively. Urine ACR: urine albumin-to-urine creatinine ratio

Figure 1 shows the study participants' serum creatinine and urine ACR levels through a scatterplot. Each of the four groups was highlighted in distinct colors. Group A and D patients had higher serum creatinine and urine ACR levels, which could be traced to their renal impairment. The remaining two groups (B and C) exhibited lower values, supporting their normal renal function. The participants in the four groups (A, B, C, and D) had correlation coefficients (between serum creatinine and urine ACR levels) of 0.23 (95% confidence interval (CI) = 0.02-0.41, p = 0.03), 0.25 (95% CI = 0.03-0.45, p = 0.02), 0.02 (95% CI = -0.23-0.26, p = 0.36), and 0.33 (95% CI = 0.07-0.55, p < 0.001), respectively.

**Figure 2:**
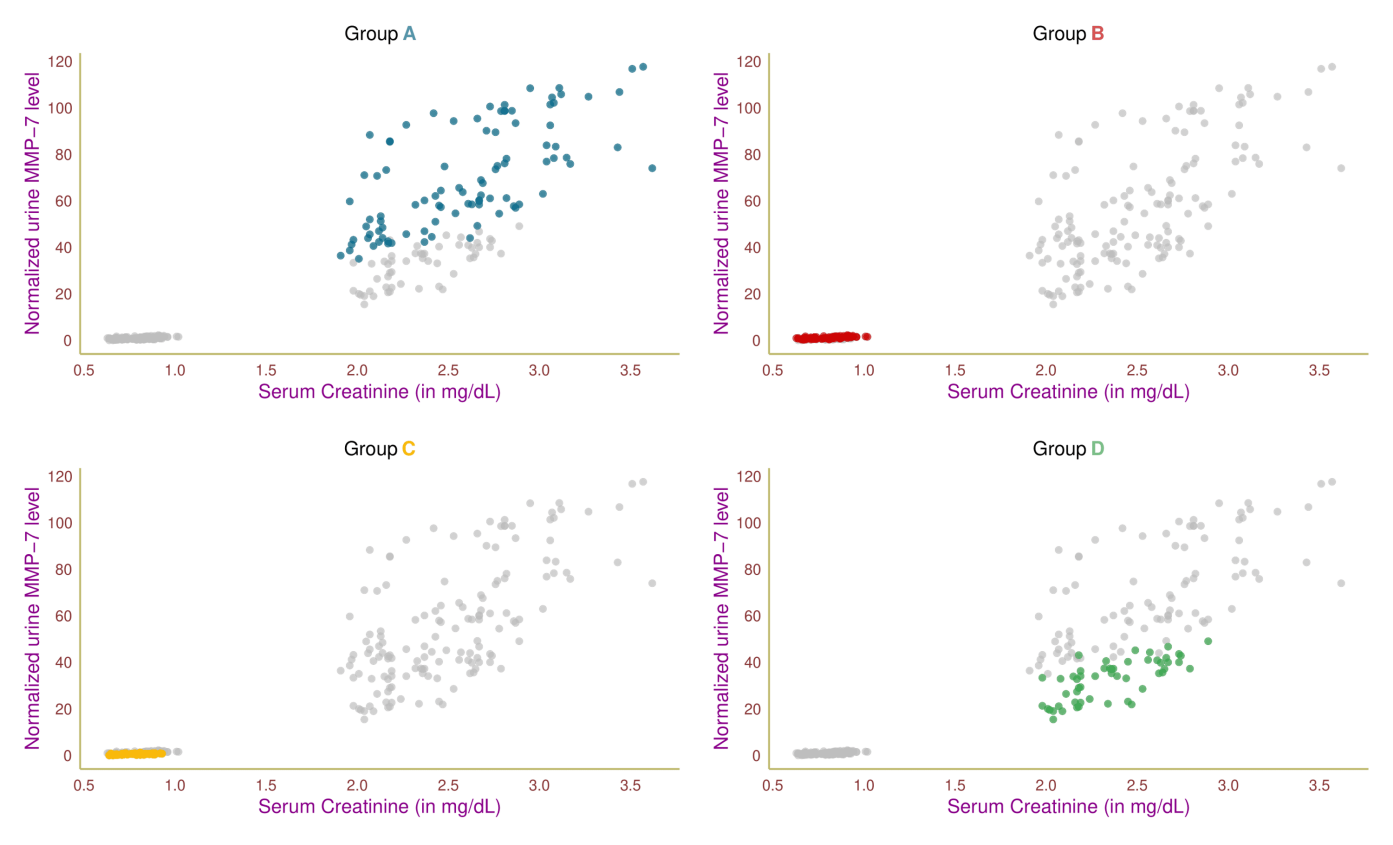
Association between serum creatinine and normalized urine MMP-7 The scatterplots showcase the association between serum creatinine and normalized urine MMP-7 values of the study participants in different colors. Groups A, B, C, and D encompassed individuals with diabetic nephropathy, diabetes only, without diabetes and nephropathy, and nephropathy only, respectively. MMP-7: matrix metalloproteinase-7, normalized urine MMP-7: urine MMP-7-to-creatinine ratio

Figure 2 shows the study participants' serum creatinine and normalized urine MMP-7 values through a scatterplot. Each of the four groups was highlighted in distinct colors. Group A and D patients had elevated levels of serum creatinine and normalized urine MMP-7 values, which could be linked to their renal dysfunction. The remaining two groups (B and C) exhibited lower values of these parameters, suggesting their normal renal function. The participants in four groups (A, B, C, and D) had correlation coefficients (between serum creatinine and normalized urine MMP-7 levels) of 0.68 (95% CI = 0.55-0.78, p < 0.001), 0.59 (95% CI = 0.42-0.72, p < 0.001), 0.51 (95% CI = 0.30-0.67, p < 0.001), and 0.72 (95% CI = 0.55-0.83, p < 0.001), respectively.

**Figure 3:**
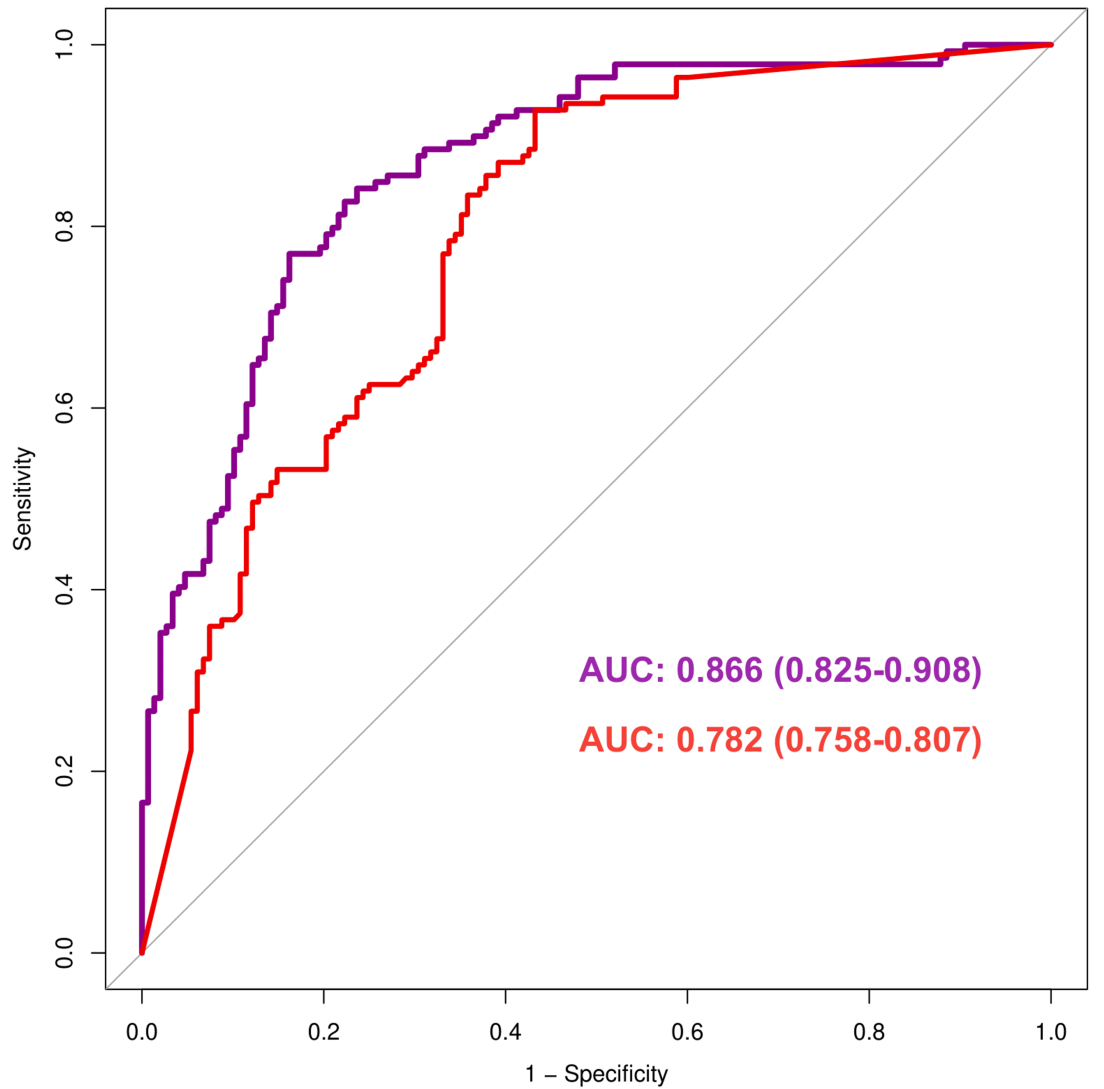
ROC curves for urine ACR and normalized urine MMP-7 The ROC curves illustrate the comparison between the sensitivity and specificity of urine ACR (shown in red color) and normalized urine MMP-7 (shown in purple color). The x- and y-axes represent 1 - specificity and sensitivity, respectively. ROC: receiver operating characteristic, AUC: area under the curve, urine ACR: urine albumin-to-urine creatinine ratio, MMP-7: matrix metalloproteinase-7, normalized urine MMP-7: urine MMP-7-to-urine creatinine ratio

The ROC curves in Figure 3 reflect the sensitivity and specificity of urine ACR (red) and normalized urine MMP-7 (purple) in predicting renal impairment in patients with and without T2DM. The 45° diagonal line (grey) depicts the points with equal true and false positive rates. The curve near this diagonal line is less accurate than nearer to the upper left corner. Furthermore, a curve with a higher AUC has greater predictive accuracy than one with a lower AUC. The sensitivity and specificity of urine ACR were 81.3% and 64.4%, respectively. The sensitivity and specificity of normalized urine MMP-7 were 86.7% and 68.2%, respectively. In this figure, the AUC for the urine ACR and normalized urine MMP-7 curves were 0.782 (0.758-0.807) and 0.866 (0.825-0.908), respectively. The graph and AUC values demonstrate that normalized urine MMP-7 provides greater predictive accuracy than urine ACR in forecasting renal impairment in people with or without T2DM. The comparison of both AUC values presented a statistically significant difference (p = 0.026).

**Figure 4:**
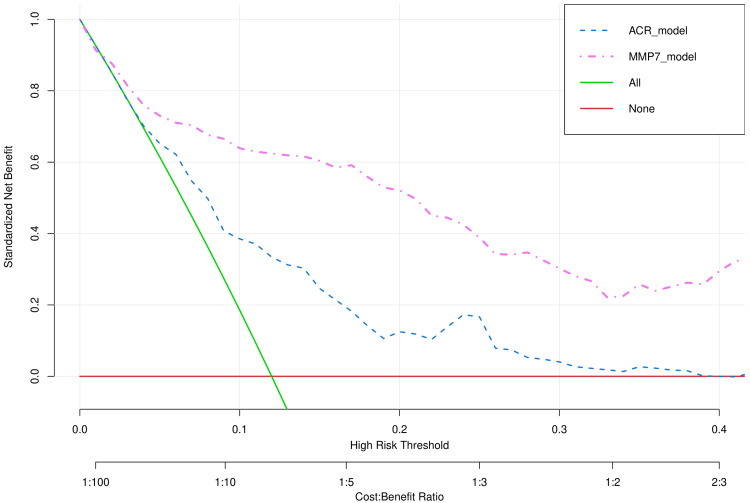
Decision curve analysis of two models predicting renal dysfunction The decision curves illustrate the two models' risk thresholds and net benefits. The x-axis represents the risk threshold, also known as the threshold probability of the disease. The x-axis also expresses the cost-benefit ratio. The y-axis represents the standardized net benefit. The ACR_model (blue dotted line) analyzes the patients with urine ACR > 30 mg/g to predict kidney function. The MMP7_model (purple dotted line) analyzes the patients with normalized urine MMP-7 > 10 µg/L to predict kidney function. ACR: albumin-to-urine creatinine ratio, MMP-7: matrix metalloproteinase-7

Figure 4 demonstrates the decision curve analysis for two models (urine ACR and normalized urine MMP-7). The x-axis shows the risk threshold, frequently designated as the disease's threshold probability. The x-axis also indicates the cost-benefit ratio. The y-axis depicts the standardized net benefit. The red line represents the net benefit of not evaluating urine ACR or normalized urine MMP-7, assuming all patients have normal kidney function. The green line represents the net benefit of evaluating urine ACR and normalized urine MMP-7, assuming all patients have impaired kidney function. The blue dotted line (i.e., ACR_model) signifies the net benefit of assessing patients with urine ACR > 30 mg/g to predict kidney function. The purple dotted line (i.e., MMP7_model) shows the net benefit of assessing patients with normalized urine MMP-7 > 10 µg/L to predict kidney function. The model with a higher benefit renders it more preferable over the other model. It is obvious from the graph that at any given threshold probability, urine MMP-7 provided more net benefit than urine ACR. The exact values of threshold probability and corresponding net benefits for the two models are provided in Table 2.

**Table 2:** Net benefits of two models at various probability thresholds The net benefits of both models for various risk thresholds (or threshold probability) were calculated. The ACR_model analyzes patients with urine ACR > 30 mg/g to predict kidney function. The MMP7_model analyzes patients with normalized urine MMP-7 > 10 µg/L to predict kidney function. ACR: urine albumin-to-urine creatinine ratio, MMP-7: matrix metalloproteinase-7

Probability threshold	ACR_model	MMP7_model
5	0.668	0.739
10	0.381	0.637
15	0.236	0.601
20	0.129	0.528
25	0.172	0.393
30	0.047	0.304
35	0.030	0.264
40	-0.001	0.301

## Discussion

In this study, we correlated urine MMP-7 and ACR with serum creatinine values of all 287 participants. Group A and D participants with impaired renal function had elevated levels of the parameters mentioned above. The remaining two groups (B and C) had those parameters within the normal range, which could be attributed to their well-functioning kidneys. The ROC curves revealed higher sensitivity, specificity, AUC, and diagnostic accuracy for urine MMP-7 than ACR. Furthermore, the decision curve analysis favored urine MMP-7 as a better predictor of renal dysfunction when juxtaposed with urine ACR.

Group A comprised 94 patients with both T2DM and chronic kidney disease (CKD). Group B had 75 diabetics who were free of kidney disease. Group C is composed of 65 individuals who had never had T2DM or kidney issues. However, they were diagnosed with either dyslipidemia or hypertension. Group D featured all nondiabetic subjects with hypertensive renal disease. Serum creatinine, urine ACR, and normalized urine MMP-7 values of group A and D subjects were substantially elevated compared to the remaining two groups' participants. However, the correlation coefficients of serum creatinine with MMP-7 values are higher than the ACR values. The facts and figures advocated that urine MMP-7 had a stronger association with serum creatinine irrespective of any sociodemographic or clinical traits. Recent studies by Tan et al. [25] and Avello et al. [26] documented stronger links between increased MMP-7 levels and the severity of renal failure. MMP-7 proved its potential as a biomarker for kidney injury. These studies [25,26] supported our observations.

Our earlier research paper demonstrated stronger associations of urine MMP-7 with serum creatinine, eGFR, and urine ACR [20]. In this article, we contrasted urine MMP-7 and urine ACR to evaluate their sensitivity, specificity, and diagnostic accuracy in forecasting and diagnosing renal impairment. We plotted ROC curves to serve the same. The ROC curves favored urine MMP-7 as a better predictive biomarker over urine ACR. Studies by Sarangi et al. [12] and Enoksen et al. [13] supported our study findings. MMPs are potentially implicated in fundamental cellular events such as angiogenesis, differentiation, migration, inflammation, and apoptosis by interacting with cell adhesion molecules, growth factors, and ECM [27]. MMPs are predominantly generated by tubular epithelial cells and glomerular intrinsic cells [12,28]. Petra et al.'s in silico analysis advocated that protein convertases and MMPs might trigger CKD-associated peptide synthesis [28].

We speculated the renal activity of our study participants through urine MMP-7 and ACR values. We constructed two predictive models, ACR_model and MMP7_model. The former model gauged the renal function of subjects with urine ACR > 30 mg/g. The later model weighed the renal function of participants with normalized urine MMP-7 > 10 µg/L. The predictive modeling leveraged the bootstrap method and decision curve analysis [29]. We discovered that regardless of the threshold probability, the MMP_7 model yielded a higher net benefit than the ACR_model. Our findings concurred with recent studies by Krochmal et al. [30] and Ihara et al. [31].

This study's primary strength was the heterogeneous study population regarding the presence of T2DM and DKD. The ROC curve and decision curve analysis were other pluses. Our study had a few limitations as well. First, the study population was from a single study site. It could hinder our study findings' generalizability. Second, we overlooked the seriousness of comorbidities or the impact of ongoing drugs. Third, we did not assess the long-term impact of renal impairment through regular follow-ups. Fourth, many factors contributing to kidney dysfunction have a lasting effect on multiple facets of quality of life. Validating each of these elements in a patient on long-term medication for T2DM, hypertension, or DKD may be difficult in practical situations. Despite all these shortcomings, we unveiled that urine MMP-7 could serve as a potential predictive biomarker for kidney injury.

## Conclusions

This study gauged the role of urine MMP-7 and ACR as potential indicators of renal dysfunction in diabetic individuals. We unveiled stronger associations of serum creatinine with urine MMP-7 juxtaposed with urine ACR. The specificity, sensitivity, and diagnostic accuracy of urine MMP-7 were higher than urine ACR. The decision curve analysis also favored urine MMP-7 over urine ACR in predicting renal impairment. We urge more research with longer study timeframes and bigger samples to figure out the long-term impact of urine MMP-7 in forecasting renal failure.
